# Acceptance and commitment therapy for psychiatric inpatients diagnosed with depression and insomnia: a multiple-baseline single-case study

**DOI:** 10.3389/fpsyt.2025.1673223

**Published:** 2026-01-05

**Authors:** Zrinka Sosic-Vasic, Max Bergmann, Julia Kroener

**Affiliations:** 1 Department of Applied Psychiatry and Psychotherapy, Christophsbad-Academy for Psychotherapy, Christophsbad Goeppingen, Goeppingen, Germany; 2 Medical Department, University of Ulm, Ulm, Germany

**Keywords:** acceptance and commitment therapy, ACT, depression, inpatients, insomnia, psychiatry, psychotherapy

## Abstract

**Introduction:**

The overarching goal of ACT is to increase psychological flexibility, which can be enhanced through mechanisms such as acceptance, valued-driven actions, or mindfulness. Several meta-analyses confirm the effectiveness of Acceptance and Commitment Therapy (ACT) in the treatment of clinical and subclinical populations, including patients diagnosed with depressive disorder and insomnia, largely within outpatient settings. However, there is no scientific research evaluating the efficacy of ACT as an adjunct treatment for psychiatric inpatients with depression. This study aimed to evaluate the efficacy of a manualized ACT treatment in addition to treatment as usual (TAU), within an inpatient setting for patients diagnosed with depressive disorder and comorbid insomnia.

**Method:**

Eight psychiatric inpatients received eight ACT sessions (two per week), in addition to TAU, which involved cognitive behavioral therapy (CBT) and psychiatric medication. A single case series design with an A-B replication across inpatients was implemented. Patients were assessed one week (T1) and one day (T2) before ACT treatment (while already receiving TAU), as well as one week (T3) and three months (T4) after treatment termination. Symptom improvement was assessed using self-report questionnaires: Beck Depression Inventory-II (BDI-II), Regensburger Insomnia Scale (RIS), Acceptance and Action Questionnaire-II (AAQ-II), Sleep Problem Acceptance Questionnaire (SPAQ), and quality of life (WHOQOL-BREF).

**Results:**

No significant change was observed from T1 to T2 (p >.05). However, significant improvement from T2 to T3 and T2 to T4 was found for depressive symptoms, insomnia, psychological flexibility, acceptance of insomnia symptomatology, and quality of life related to physical and psychological symptoms.

**Discussion:**

The findings suggest that ACT is a promising adjunct intervention, applicable transdiagnostically for individuals suffering from depression and comorbid insomnia in inpatient settings. By enhancing psychological flexibility and symptom acceptance, ACT may offer unique benefits beyond traditional CBT, particularly for patients with residual symptoms, chronic distress, or recurrent depressive episodes. Despite these promising findings, results must be interpreted with caution due to the small sample size and lack of a control group. Future research should replicate and extend these findings in larger randomized controlled trials to further evaluate ACT’s potential in inpatient psychiatric care.

## Introduction

Depressive disorders are among the most prevalent psychiatric disorders worldwide, with detrimental effects on the individual´s well-being, as well as significant impacts on public health concerns. According to the World Health Organization (WHO), there are about 332 million people globally, who suffer from depressive disorders (1). Looking at high-income countries, lifetime prevalence ranges between 10% and 15%, with higher prevalence rates in women as compared to men (2). Depressive disorders are associated with considerable functional impairment (e.g., decreased work performance, low marital quality), overall reduced quality of life, elevated risks for the development-, and severity of secondary disorders (such as physical disease), as well as increased mortality rates, particularly due to a heightened risk for suicide (2, 3). Given the high prevalence, burden of disease, and the tremendous associated healthcare and economical costs (4–7) the development of effective and feasible treatment programs remains an essential part of clinical research.

Within the context of depressive disorders, there is substantial evidence for an association between depressive symptoms and insomnia. For example, past research suggest that approximately 75% of individuals diagnosed with major depressive disorder (MDD) report clinically significant levels of sleep disturbance (8, 9). Moreover, while insomnia is a common symptom of depression, it proves also to be a risk factor for its onset, chronicity, and reoccurrence (10–12). The bidirectional relationship between depression and insomnia (13) suggests that effective treatment programs should emphasize both conditions simultaneously. The finding that chronic sleep disturbances in individuals diagnosed with depression have been linked to poorer treatment outcomes, increased relapse rates, and increased levels of suicidal ideation (14) further supports this notion. Considering these scientific findings, interventions that simultaneously target depressive symptoms and associated sleep disturbances warrant further examination.

As depressive disorders are oftentimes associated with an increased risk for suicide, functional impairment, or acute symptom acerbation, there is a heightened demand for inpatient psychiatric treatment within this population. Specifically, 1.3% of all hospitalizations in the U.S. are primarily due to major depressive disorders (MDD; 15). Recent longitudinal data from inpatient and day-hospital settings indicate that only about one-third of patients achieve sustained remission during the first year after treatment; many follow trajectories characterized by relapse, temporary relapse, or recurrence (16). In general psychiatric settings, readmission rates of 33% at three months and up to 41% at one year have been reported (17). Among individuals with MDD, even after guideline-concordant treatment, roughly 50% relapse or recur after a first episode (18). Furthermore, conventional inpatient treatments, including pharmacotherapy and psychotherapy, often focus on the primary diagnosis at hand, hence missing to address comorbid conditions such as insomnia effectively. Henceforth, identifying transdiagnostic psychotherapeutic approaches that enhance treatment response, remission, and long-term recovery in inpatients with depression and comorbid symptoms—such as insomnia—appears crucial.

Inpatient psychiatric treatment for patients diagnosed with depression typically involves a multimodal approach, including pharmacotherapy, psychotherapy, and various forms of functional treatments (e.g., arts therapy, occupational therapy). While selective serotonin reuptake inhibitors (SSRIs) and other antidepressants remain first-line pharmacological treatments (19), response rates are insufficient, with approximately one-half to two-thirds of patients failing to achieve remission (20). Psychotherapeutic interventions — especially Cognitive-Behavioral Therapy (CBT) — have shown effectiveness in reducing depressive symptoms not only in research trials but also in routine clinical care, including inpatient or day-clinic settings (21–23). However, recent reviews and naturalistic studies indicate that effect sizes tend to be small to moderate when considering inpatient psychotherapy against standard care (i.e., TAU), and evidence remains limited by methodological heterogeneity, varying treatment formats, and often small sample sizes (21, 22, 24). As a result, while CBT continues to be a central pillar of psychotherapeutic in-patient care, the enthusiasm for very large or long-lasting effects must be seen with caution, especially in severely ill, comorbid or treatment-resistant populations. In turn, newer approaches — including integrated or modified CBT, acceptance- and mindfulness-based therapies, and combined psychosocial interventions — have gained increased attention over the past decade. Their transdiagnostic flexibility makes them attractive for patients with complex clinical presentations (e.g., comorbid depression and insomnia), and they may complement or in part address limitations of standard CBT in inpatient settings. Specifically Acceptance and Commitment Therapy (ACT), has proven to be a promising alternative or addition to existing therapeutic approaches within the treatment of depressive disorders (e.g., 25).

Acceptance and Commitment Therapy (ACT) is a contextualistic therapeutic approach rooted within CBT and the Relational Framework Theory (RFT) that aims to enhance psychological flexibility by fostering being in contact with the present moment, while acting in accordance with one´s values (e.g., 26). Unlike traditional forms of CBT, which focus on modifying dysfunctional or maladaptive thoughts, ACT targets cognitive fusion as well as experiential avoidance, and encourages individuals to relate to their thoughts and emotions in a non-judgmental, mindful, and accepting manner, while committing to meaningful and value-driven life, despite the presence of psychological distress. To achieve this goal, ACT incorporates six interrelated core processes—cognitive defusion, acceptance, mindfulness, self-as-context, values clarification, and committed action—each contributing individually to the construct psychological flexibility (e.g., 27). By shifting the therapeutic focus from symptom reduction and a problem solving to a mindset that promotes a more open, centered, and engaged approach to living, ACT may provide a valuable alternative for individuals struggling with chronic and treatment-resistant forms of depression.

ACT has proven to be an effective psychotherapeutic treatment for various psychiatric disorders, including anxiety disorders (e.g., 28), post-traumatic stress disorder (PTSD; 29), obsessive-compulsive disorder (OCD; 30), and psychosis (e.g., 31). Meta-analyses indicate that ACT is at least as effective as standard cognitive-behavioral therapy, with unique benefits in reducing experiential avoidance, enhancing psychological well-being, and increasing psychological flexibility (e.g., 32, 33). Given its transdiagnostic applicability and emphasis on acceptance-based therapeutic strategies, ACT seems particularly applicable for individuals with comorbid psychiatric conditions, such as depression and insomnia, which often involve high levels of distress intolerance and cognitive rumination (e.g., 34, 35).

Looking at the treatment of depressive disorders specifically, past research has provided evidence supporting the effectiveness of ACT in treating unipolar depression (e.g., 36). Various randomized controlled trials (RCTs) have demonstrated that ACT is effective in reducing depressive symptoms, decreasing experiential avoidance, and improving psychological flexibility (for a meta-analysis see 25) been found to be particularly beneficial for individuals with chronic or recurrent depression, as this treatment approach focuses on reducing rumination and enhances the engagement in meaningful valued-driven activities (e.g., 37). Within inpatient settings, however, research on the efficacy and effectiveness of ACT for depression remains limited. Initial studies show that ACT-based interventions can be feasibly implemented in psychiatric hospital settings and may contribute to improved treatment outcomes in patients with depression, even in the presence of severe comorbid disorders, such as substance abuse (e.g., 38, 39). Moreover, ACT’s emphasis on mindfulness and acceptance-based strategies may be particularly applicable for addressing insomnia-related difficulties in patients with depression, yet little research has specifically examined this application. However, within the context of ACT as a treatment for insomnia, there has been promising evidence for its effectiveness (e.g., 40, 41).

Despite the growing body of evidence supporting ACT as a viable and beneficial treatment for depression and insomnia separately, there are still several research gaps that need to be addressed. First, the vast majority of studies on the treatment effects of ACT have been conducted within outpatient settings, resulting in a lack of knowledge about the treatment´s effectiveness in inpatient psychiatric care. Second, while ACT has demonstrated promising effects in the treatment of sleep disturbances, research investigating its impact on insomnia symptoms in patients with comorbid depression is scarce. Given these gaps within the scientific literature, methodologically rigorous studies examining the feasibility, acceptability, and efficacy of ACT in psychiatric inpatient settings are warranted, particularly for individuals with depression and comorbid insomnia. Therefore, the present study aims to investigate the efficacy of ACT for psychiatric inpatients diagnosed with unipolar depression and comorbid insomnia as an adjunct treatment to treatment-as-usual (TAU) using a multiple-baseline single-case study design. Specifically, the study aims to explore (1) whether ACT leads to improvements in depressive symptoms, (2) whether ACT contributes to a reduction in insomnia severity, and (3) whether ACT increases psychological flexibility, acceptance of sleep related problems, and quality of life. By employing a single-case multiple-baseline approach, this study provides a nuanced analysis of individual treatment responses, compares treatment effects with-, and without adjunct ACT treatment sessions, and contributes to the growing evidence evaluating ACT in inpatient psychiatric settings. Findings from the current study may inform the development of future transdiagnostic ACT treatment programs for hospitalized patients, possibly enhancing treatment outcomes and long-term remission rates.

## Materials and methods

### Design

The study utilized a single case series design with an A-B replication across patients, incorporating follow-up measures (42). Patients were allocated to a baseline period of one week with only receiving the inpatient treatment as usual and without receiving the ACT-based add-on intervention. This procedure was implemented to establish individual baselines that may serve as control periods. There were four measurement time-points across the study (T0 = 1 week before first individual ACT-therapy session, T1 = one day before first individual ACT-therapy session, T2 = one week after last individual ACT-therapy session, and T3 = three months after last individual ACT-therapy session). Patients received reminders per e-mail to complete questionnaires, and were able to complete all assessments online via SoSciSurvey.

### Participants

Ten patients diagnosed with severe depressive disorder and comorbid symptoms of insomnia were included within the study. However, one patient had to be excluded due to receiving electroconvulsive therapy during inpatient treatment, and one patient had to be excluded following the confirmation of a suspected positional obstructive sleep apnoea syndrome as well as due the initiation of electroconvulsive therapy during the follow-up assessment period. Therefore, eight patients diagnosed with severe depressive disorder and comorbid symptoms of insomnia (5 females & 3 males) were analyzed within this study. The mean age was 52 years (*SD* = 10.3; see **Table 1** for patient characteristics). All subjects (*N* = 8) were recruited via the depression unit PSY4 of the Christophsbad Clinic located in Goeppingen (Germany). Recruitment took place between July 2023 and February 2024. Patients were briefly screened for inclusion and exclusion criteria and invited for a diagnostic interview thereinafter. At the diagnostic interview, patients received general information about the study and the study setting, and provided written informed consent. Afterwards, the M.I.N.I. International Neuropsychiatric Interview (43) was conducted to assess psychiatric comorbidity. Furthermore, the section A of the SCID-5-CV interview (44) was implemented to assess for symptoms of depression and insomnia. The Beck Suicidal Ideation Scale was used to assess suicidality. The study was conducted in accordance with the Declaration of Helsinki, and approved by the Ethics Committee of the Medical Board Baden-Wuerttemberg.

**Table 1:** Patient characteristics.

	Patient 1	Patient 2	Patient 3	Patient 4	Patient 5	Patient 6	Patient 7	Patient 8
Age	55-60	60-65	35-40	55-60	55-60	30-35	50-55	55-60
Sex	female	female	female	female	male	male	female	male
Marital status	married	married	married	married	married	in a committed partnership	in a committed partnership	married
Education level	secondary school certificate	secondary school certificate	advanced school-leaving certificate	secondary school certificate	secondary school certificate	secondary school certificate	secondary school certificate	secondary school certificate
Comorbid psychiatric diagnoses	none	none	Attention-Deficit/Hyperactivity Disorder (ADHD), Predominantly Inattentive Presentation	none	none	none	Borderline Personality Disorder (BPD)	none
Psychotherapy (in the past)	none	none	none	outpatient therapy (1x)	none	none	inpatient therapy (2x)	inpatient therapy (2x)
Inpatient Psychopharmacological treatment	first Milnacipran, then switch to Sertraline, Quetiapine	Lorazepam, Mirtazapine, Sertraline	Bupropion, Quetiapine, Methylphenidate	Sertraline, Quetiapine	Duloxetine, Quetiapine	Sertraline, Mirtazapine, Quetiapine, Trazodone	Sertraline, Quetiapine, Pipamperone	Duloxetine, Quetiapine

Inclusion criteria were: (a) diagnosis of a current depressive episode according to ICD-10 criteria (F32.0–F32.2 or F33.0–F33.2), assessed during the diagnostic intake session; (b) diagnosis of insomnia disorder (ICD-10 F51.0), including difficulties initiating or maintaining sleep and early morning awakenings with the inability to fall back asleep; (c) minimum age of 18 years; and (d) fluent proficiency in German, both spoken and written, to ensure comprehension of diagnostic assessments and the intervention.

Exclusion criteria were: (a) intellectual disability based on clinical impression; (b) acute suicidality; (c) acute risk of harm to others; (d) acute self-injurious behavior; (e) acute psychotic symptoms; (f) current or past episodes of bipolar disorder; (g) current or past diagnoses within the schizophrenia spectrum; (h) presence of a substance addiction; (i) diagnosis of hypersomnia; (j) suspected or diagnosed obstructive sleep apnoea syndrome; and (k) current biological treatment methods, including electroconvulsive therapy (ECT) or esketamine treatment (Spravato).

### Measures

*Beck Depression Inventory II* (BDI-II; 45). The BDI-II is a self-report questionnaire used to assess the severity of depressive symptoms. It consists of 21 items, each rated on a 4-point Likert scale ranging from 0 to 3, with higher scores indicating more severe depressive symptomatology. The internal consistency of the BDI-II has been reported as good (Cronbach’s α = .88; 46).

*Acceptance and Action Questionnaire II* (AAQ-II; German Version: 47). The FAH-II is the German adaptation of the Acceptance and Action Questionnaire (AAQ-II). It is a change-sensitive self-report measure designed to assess psychological flexibility, particularly in the context of ACT interventions. The questionnaire consists of 7 items evaluating the acceptance of psychological symptoms and the ability to take action despite their presence. The internal consistency of the AAQ-II is reported to be good to excellent, with a Cronbach’s α of.84 in patients with social anxiety disorder and.97 in student samples (47).

*Regensburg Insomnia Scale* (RIS; 48). The RIS is a self-report questionnaire designed to assess cognitive, emotional, and behavioral aspects of psychophysiological insomnia. Originally developed to evaluate the effectiveness of cognitive-behavioral therapy for insomnia (CBT-I), the scale consists of 10 items. It has demonstrated strong internal consistency, with a Cronbach’s α of.89 (48).

*Sleep Problem Acceptance Questionnaire* (SPAQ; 49). The SPAQ is a self-report measure assessing the acceptance of sleep problems. It consists of eight items and is primarily used in research on the effectiveness of Acceptance and Commitment Therapy (ACT) for insomnia. The questionnaire comprises two subscales with four items each: Activity Engagement and Willingness. The internal consistency of both subscales was reported as satisfactory in the original study, with Cronbach’s α values of.89 for Activity Engagement and.73 for Willingness. The Cronbach’s α for the scale as a whole was lower, at.55 (49).In this study, a German version of the SPAQ is used, which was developed through a certified translation service.

*WHO Quality of Life Questionnaire - Short Version* (WHOQOL-BREF; 50). The WHOQOL-BREF is the short version of the WHOQOL-100 and consists of 26 items covering four domains: physical health, psychological well-being, social relationships, and environment. Additionally, two items assess global quality of life. The WHOQOL-BREF has demonstrated good reliability and validity (50), with normative reference values available for different age groups (51). According to the German test manual, the internal consistency (Cronbach’s α) of the WHOQOL-BREF subscales ranges from α = .57 to α = .88, indicating acceptable to good reliability (52).

*Patient satisfaction questionnaire (self-developed).* At T2, patient satisfaction was assessed using a self-developed questionnaire. The aim was to capture various dimensions of patient satisfaction. The questionnaire included 10 items and utilized a 10-point Likert scale (1 = not at all to 10 = very strongly). Participants were asked to rate their satisfaction with the treatment received, as well as the benefits gained from the treatment. Examples of items included: “I am satisfied with the treatment I have received” and “I have benefited from the treatment.” Additionally, there was an option for patients to provide free-text feedback on their experiences.

### Intervention

To enhance clarity, the interventions are presented in two separate subsections. First, we describe the standard inpatient treatment as usual (TAU), which all participants received as part of routine care. Second, we outline the ACT-based add-on intervention, which was delivered in addition to TAU and represents the experimental component of this study.

#### Treatment as usual

The treatment as usual (TAU) provided on the depression unit PSY4 at Christophsbad Clinic Göppingen comprises a multimodal, interdisciplinary therapeutic program characteristic of inpatient psychiatric care. TAU includes psychopharmacological treatment as well as individual cognitive-behavioral therapy (CBT)-oriented psychotherapy with an assigned therapist, delivered either once weekly (50 minutes) or twice weekly (25 minutes). These individual sessions typically incorporate behavioral activation, cognitive restructuring, activity scheduling, problem-solving strategies, and elements of mindfulness-based stress regulation, depending on clinical needs. In addition to individual therapy, patients participate in several structured behavioral therapy group formats, including psychoeducation, social skills training, and open discussion groups. The inpatient program also integrates physical activity interventions such as morning exercise, gymnastics, yoga, and hiking, as well as creative therapies including art therapy, music therapy, and occupational therapy. This multimodal program targets broad symptom domains—affect regulation, behavioral activation, cognitive flexibility, interpersonal functioning, and physical well-being—providing a comprehensive therapeutic framework into which the ACT-based add-on intervention was embedded.

#### ACT-based add-on therapy

The ACT-based add-on intervention consisted of eight manualized individual sessions, delivered twice weekly over a four-week period during the inpatient stay. Each 50-minute session was structured to ensure consistent implementation of Acceptance and Commitment Therapy principles (53) and to allow individualized adaptation to depressive and insomnia-related symptoms. Metaphors, experiential exercises, and mindfulness-based practices were selected from established ACT manuals (54, 55).

Sessions followed a standardized sequence. Each meeting began with a brief check-in to assess the patient’s current emotional state, followed by a review of homework assignments from the previous session (except in Session 1). The therapist then introduced the session’s central metaphor or experiential exercise and provided a brief conceptual or psychoeducational component to deepen understanding of ACT processes. The core of each session involved practical application of ACT techniques tailored to the patient’s difficulties with depression and insomnia. Sessions concluded with a summary and the introduction of a new homework assignment (except in Session 8). **Table 2** provides an overview of session content and thematic progression.

**Table 2:** Session content.

Session	Contents
1	Introduction to Acceptance and Commitment Therapy (ACT), therapeutic rationale, rapport building, and creative hopelessness in the context of depression and insomnia.
2	Introduction and exploration of the therapy component Mindfulness and Present-Moment Awareness in the context of depression and insomnia.
3	Introduction and exploration of the therapy component Acceptance and Willingness in the context of depression and insomnia.
4	Introduction and exploration of the therapy component Cognitive Defusion in the context of depression and insomnia.
5	Introduction and exploration of the therapy component Self-as-Context in the context of depression and insomnia.
6	Introduction and exploration of the therapy component Values in the context of depression and insomnia.
7	Introduction and exploration of the therapy component Committed Action in the context of depression and insomnia
8	Conclusion of therapy and relapse prevention

### Statistical analysis

The multiple baseline-single case series design involved visual inspection of data for each patient to assess treatment effects. This procedure facilitates the evaluation of individual changes over time and the assessment of each patient’s range and stability of change. Nonetheless, the sole assessment of descriptive data may lead to a Type I error. Subsequently, outcome measures were analyzed utilizing percentage values. The treatment response, operationalized as a 30% reduction in depressive symptoms at post-treatment (T2), was assessed for each patient. Furthermore, reliable change was evaluated for each patient using the Reliable Change Index (RCI) as established by Bauer et al. (56). This assessment utilized standard deviations and alpha coefficients from a prior study including adults diagnosed with depression (57), and focused on clinical outcome measures related to depressive symptoms, specifically the Beck Depression Inventory-II (BDI-II). An RCI of 1.96 or greater indicates a significant change. Additionally, paired sample t-tests were conducted at all measurement time points (T0, T1, T2, FU) to evaluate changes within the multiple baseline time frame (T0 to T1) and from pre-treatment (T1) to post-treatment (T2) and follow-up (FU) for the overall group. Effect sizes from pre- to post-intervention and follow-up were computed using Cohen’s d (58).

## Results

### Changes in depressive symptoms, insomnia, and acceptance of sleep related problems

**Figure 1:**
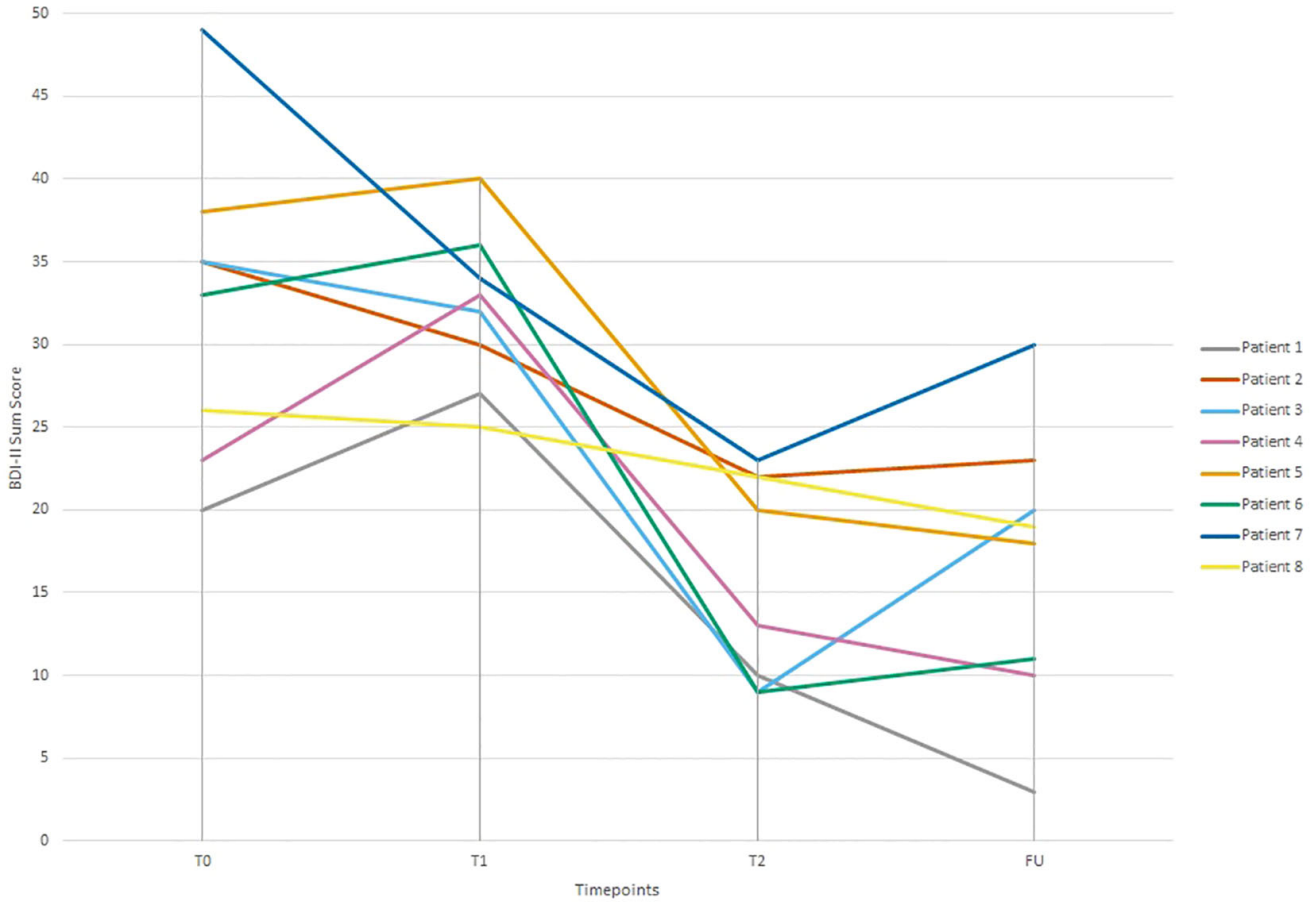
Beck-Depression Inventory II (BDI-II) sum scores across time-points (T0 = one week before first therapy session; T1 = one day before first therapy session; T2 = one week after last therapy session; FU = three months after last therapy session) and patients. Depressive symptoms improved for all patients from T0 to FU.

**Table 3** presents scores for all patients, measurements and measurement time-points. Looking at depressive symptoms, there was a significant 50% reduction of depressive symptoms one week after treatment termination, t(7) = 5,62, p ≤ 0.001, d = 1.99. Moreover, there was a significant 48% reduction in depressive symptoms three months after treatment termination, t(7) = 4.83, p ≤ 0.001, d = 1.71 for the group as a whole, demonstrating that gains were maintained during the follow-up period. Six out of eight patients met criteria for being treatment responders (≥ 30% reduction in depressive symptoms at post-treatment), whereby 5 out of 8 patients (1, 3, 4, 5, 6) revealed RCIs greater than 1.96 at post-treatment as well as follow-up, indicating significant changes in depressive symptomatology. Two patients (patients 2 and 8) were improving symptomatically; however, improvements were < 30% at post-treatment and follow-up. Moreover, patient 7 was considered a treatment responder at post-treatment, however, symptom improvement decreased below 30% at follow-up. Changes in depressive symptoms for each patient are shown in **Figure 1**.

**Table 3:** Outcome measures across time and patients.

	Timepoints	Patient 1	Patient 2	Patient 3	Patient 4	Patient 5	Patient 6	Patient 7	Patient 8	Mean (*SD*)	Effect size *d^1^*
Depressive Symptoms (BDI-II)	T0	20	35	35	23	38	33	49	26	32,38(9,29)	
	T1	27	30	32	33	40	36	34	25	32,13(4,82)	0,032
	T2	10	22	9	13	20	9	23	22	16,00(6,33)	1,99***
	FU	3	23	20	10	18	11	30	19	16,75(8,45)	1,71***
% reduction of depressive sx	T1-T2	62,96	26,67	71,88	60,61	50,00	75,00	32,35	12,00	50,20	
	T1-FU	88,89	23,33	37,50	69,70	55,00	69,44	11,76	24,00	47,87	
Insomnia (RIS)	T0	31	21	25	17	30	10	38	25	24,63(8,73)	
	T1	26	22	24	24	29	13	33	23	24,25(5,80)	0,093
	T2	26	21	11	22	17	12	30	22	20,12(6,53)	0,79*
	FU	10	16	14	17	19	10	22	21	16,13(4,58)	1,77***
% reduction of insomnia sx	T1-T2	0,00	4,54	54,17	8,33	41,38	7,69	9,09	4,35	17,03	
	T2-FU	61,54	27,27	41,67	29,17	34,48	23,08	33,33	8,70	33,48	
AAQ-II	T0	17	27	35	33	40	25	41	36	31,75(8,19)	
	T1	23	25	32	32	43	30	40	33	32,25(6,76)	0,14
	T2	11	25	9	30	36	34	36	39	27,50(11,63)	0,51
	FU	12	14	20	10	30	35	36	37	24,25(11,50)	0,88*
SPAQ	T0	9	21	19	20	14	11	25	18	17,13(5,38)	
	T1	7	24	22	11	20	10	15	14	15,38(6,09)	0,30
	T2	18	33	37	25	18	15	11	23	22,50(8,91)	1,02*
	FU	27	32	28	31	24	13	14	21	23,75(7,25)	1,10**
WHO QOL-BREF Physical Sx	T0	21	25	36	50	50	29	39	36	35,75(10,66)	
	T1	14	32	14	43	39	32	54	46	34,25(14,43)	0,12
	T2	36	43	54	50	61	50	43	54	48,88(7,90)	0,99*
	FU	43	32	36	64	61	43	46	46	46,38(11,10)	0,91*
WHO QOL-BREF Psychological Sx	T0	46	38	50	54	33	46	29	46	42,75(8,60)	
	T1	58	42	46	46	29	46	38	50	44,38(8,52)	0,24
	T2	67	42	58	50	67	42	46	46	52,25(10,43)	0,58
	FU	67	54	54	63	50	42	38	54	52,75(9,66)	1,00*

SD, standard deviation; sx, Symptoms; BDI-II, Beck Depression Inventory-II; RIS, Regensburg Insomnia Scale; AAQ-II, Acceptance and Action Questionnaire-II; SPAQ, Sleep Problem Acceptance Questionnaire; WHO QOL-BREF, WHO Quality of Life Questionnaire-Short Version; ^1^Effect sizes were calculated from T0 to T1 (T1), T1 to T2 (T2) and from T1 to FU (FU) using Cohens *d.*

* = p < .05; ** = p < .01; *** = p < .001.

Regarding insomnia symptomatology (RIS), there was a significant 17% symptom reduction one week after the intervention, t(7) = 2.22, p ≤ 0.05, d = 0.79, as well as a significant 33% reduction in the latter insomnia symptoms three month after the intervention, t(7) = 5.02, p ≤ 0.001, d = 1.77, demonstrating that further gains were made during the follow-up period. Concerning the acceptance of sleep related problems, as assessed by the SPAQ, there was a significant 46% increase in acceptance scores for the group as a whole one week after treatment termination, t(7) = -2.88, p ≤ 0.05, d = 1.02, as well as a 54% increase in acceptance of sleep related problems three months after treatment termination, t(7) = -3.08, p ≤ 0.01, d = 1.10, demonstrating that further gains were made during the follow-up period.

### Changes in psychological flexibility, physical wellbeing, and psychological wellbeing

Looking at psychological flexibility, there was a non-significant 15% increase (AAQ-II) one week after treatment termination, t(7) = 1.44, p = 0.097, as well as a 30% increase three months after treatment termination, t(7) = 2.48, p ≤ 0.05, d = 0.88, signifying that further gains were achieved during the follow-up period.

For physical well-being (WHO QOL-BREF Physical Symptoms), there was a significant 43% increase in well-being one week after treatment termination t(7) = -2.79, p ≤ 0.05, d = 0.99, as well as a significant 35% increase three months after treatment termination, t(7) = -2.56, p ≤ 0.05, d = 0.91, indicating that symptom improvement was largely maintained during the follow-up period.

Regarding psychological well-being (WHO QOL-BREF Psychological Symptoms), there was a non-significant 18% increase from pre-treatment to post-treatment, t(7) = -1.64, p > 0.05. However, psychological well-being significantly increased about 19% three months after treatment termination, t(7) = -2.83, p ≤ 0.05, d = 1.00, demonstrating that further symptom improvement was achieved during the follow-up period.

### Feasibility, acceptability, and adverse events

Recruitment procedures were successful. The target of recruiting 10 patients with a diagnosis of depression with comorbid insomnia was achieved within six months after study initiation. Nine out of ten patients recruited to participate in the study, who met inclusion criteria, completed all ACT treatment sessions as well as assessment time-points. Two patients had to be excluded from the study due to receiving ECT either during their ongoing inpatient stay, or during the follow-up assessment period. No adjustments had to be made to the study protocol.

Furthermore, treatment acceptability (self-developed questionnaire) was high for all included patients, ranging between 7.00 and 9.67 points (*M* = 8.64, *SD* = 0.91). Specifically, patients reported that they have felt understood by their therapist (*M* = 9), and that they benefitted from the treatment program (*M* = 9). Furthermore, patients indicated that they would recommend the treatment program to others with similar problems (*M* = 9), and that they have gained new insights into their symptomatology (*M* = 10).

Suicidality as an indicator for adverse events was monitored across treatment using the BSS. Suicidality declined from pre-treatment (*M* = 2.00, *SD* = 2.00) to post-treatment (*M* = 0.50, *SD* = 1.07), well as from pre-treatment to follow-up (*M* = 0.88, *SD* = 1.81).

## Discussion

The present study examined the efficacy of an Acceptance and Commitment Therapy (ACT) intervention for inpatients with a diagnosis of severe depressive disorder and comorbid symptoms of insomnia, in addition to treatment as usual (TAU). The results display clinically significant improvements across several psychiatric domains: depressive symptom severity, insomnia-related symptoms, acceptance of sleep problems, psychological flexibility, and various facets of well-being, such as physical and psychological well-being. Notably, depressive symptoms decreased across all included patients by 50% at post-treatment, as well as by 48% at the three-month follow-up. On a similar note, improvements were noted for insomnia symptoms and sleep problem acceptance. For instance, sleep related problems decreased on average by 17% at post-treatment. Although gains in psychological flexibility were moderate and non-significant at post-treatment, significant improvements emerged during the follow-up period. Moreover, significant improvements in physical and psychological well-being at post-treatment as well as follow-up provide further evidence supporting the ACT intervention´s benefits within the treatment of depressive disorders and comorbid insomnia, signifying it´s potential application as a transdiagnostic intervention. These findings contribute to the increasing literature on the advantages of ACT for the treatment of depressive disorders—specifically within an inpatient context—and highlight its ability to mitigate comorbid symptoms of insomnia by targeting mechanisms such as experiential avoidance and cognitive fusion.

Looking at the findings concerning symptoms of depression, a marked reduction—as demonstrated by a significant 50% decrease one week after treatment termination, as well as a 48% decrease at follow-up three months post-intervention—further supports previous research on the efficacy of ACT interventions for the treatment of severe depressive disorders within an inpatient setting (39, 59). The large effect sizes (d > 1.7) evolving around the reduction of depressive symptoms across measurement time-points further signify that ACT provides a meaningful contribution to symptom reduction in a clinical population well-known to have high baseline symptom severity scores, as well as various comorbid psychiatric disorders (60, 61). In addition, six out of eight patients met the criteria for being treatment responders, with reliable change indices (RCIs) affirming that these reductions are not attributable to measurement variability. The here presented preliminary findings are aligning with previous studies that revealed the clinical utility of ACT for patients with depression within outpatient settings (25), and extend our understanding by demonstrating similar benefits for psychiatric inpatients, a treatment context in which patients are at high risk for rapid relapse and severe symptomatology (62).

Regarding sleep related disturbances, the current study’s results indicated a 17% reduction in insomnia symptoms one week after treatment termination, and an even more pronounced 33% symptom reduction at the three-month follow-up, including large effect sizes (d >.79) at both measurement time-points, underscoring the benefits of ACT-based interventions within the treatment of symptoms commonly experienced in insomnia disorder. These symptomatic improvements are further supported by significant increases in the acceptance of sleep-related problems (46% immediately post-treatment and 54% at follow-up; d > 1.02). Looking at specific features of ACT, the incorporation of mindfulness exercises and experiential techniques may facilitate a shift in the patients´ sleep-related anxieties, ultimately reducing hyperarousal and maladaptive cognitive processes, such as rumination, that perpetuate insomnia (63). This bidirectional benefit (i.e., targeting depressive- and insomnia symptoms simultaneously) supports the notion that ACT can be applied transdiagnostically, which is consistent with previous research that hypothesizes that both psychiatric conditions are rooted in experiential avoidance (64–66).

With respect to psychological flexibility, post-treatment results displayed solely a non-significant 15% increase (as measured by the AAQ-II). However, there was a significant 30% increase in psychological flexibility at three-month follow-up displaying a large effect size (d = 0.88), which indicates that the psychological processes targeted during ACT may require a certain time-period to consolidate. Psychological flexibility, the overarching construct within ACT’s framework, reflects on the individual’s capability to remain mindful in the present moment while acting in accordance with one´s values, despite the possible presence of emotional distress. Within the current study, it could be hypothesised that the patients´ delayed yet significant improvement in psychological flexibility could be due to a gradual integration of the skills learned during treatment, thus resulting in the development of more adaptive behavioral patterns over time. Aligning with this notion, there were also significant improvements in both, physical well-being (43% increase at post-treatment and 35% at follow-up) as well as psychological well-being (a non-significant 18% improvement at post-treatment that reached a 19% significance at follow-up) over time. These findings suggest that ACT may offer broader benefits on mental and physical health than mere symptom reduction, a hypothesis that aligns with previous findings (67–69). It could be assumed that increased physical and psychological quality of life result from improvements in mood, sleep, and an enhanced capacity to engage in everyday activities, which could in turn potentially reduce the overall burden of disease associated with chronic depression. Furthermore, improvements in the acceptance of sleep-related problems, as displayed within the current study, could help patients to further adopt a non-judgmental stance towards distressing internal experiences, which may be a key mechanism underlying symptom improvement. This notion could be investigated within future research by conducting mediator analysis that assess sleep related problems, the acceptance thereof, as well as symptom improvement. From a clinical stance, this shift in perspective—from attempting to eliminate unwanted thoughts and feelings to learning to coexist without having to engage or act upon them—may reduce associated secondary distress that routinely contributes to the exacerbation of depressive and insomnia symptoms. The delayed improvement in psychological flexibility and psychological quality of life observed in the present study might be reflective of the time required for such a cognitive-emotional shift to take place, thus offering a possible explanation as to why improvements in certain domains were more pronounced at three-month follow-up.

The above presented findings have several meaningful implications for clinical practice. First, implementing ACT as an adjunct treatment to TAU within an inpatient setting appears to enhance treatment outcomes for patients diagnosed with acute psychiatric conditions and associated comorbidities, such as depression and insomnia. The observed benefits in both, affective and sleep-related psychiatric conditions, suggest that ACT could be effectively integrated into pre-existing multimodal inpatient treatment programs, targeting transdiagnostic symptoms. This integration could be specifically crucial within inpatient settings, where patients experience high relapse rates, and comorbid psychiatric conditions, as the present study demonstrates preliminary evidence for ACT´s transdiagnostic application, and long-term benefits. By targeting transdiagnostic psychological phenomena, such as experiential avoidance, cognitive fusion, or rumination, ACT may serve as a flexible tool for clinicians to address multiple symptom domains at once. To further investigate the possible benefits of ACT in inpatient settings, future research could focus on conducting large-scale randomized controlled trials including a TAU and a TAU with adjacent ACT treatment, as well as comparing ACT-enhanced treatment with other transdiagnostic interventions, such as mindfulness-based stress reduction (MBSR). Future RCTs should compare ACT as a standalone intervention with two alternative conditions: (1) TAU alone (e.g., CBT-oriented inpatient care), and (2) a combined ACT–CBT condition to evaluate potential synergistic mechanisms. Such dismantling studies would clarify whether ACT confers benefits beyond standard CBT or whether additive or synergistic effects account for improvements. Moreover, such RCTs should include not only self-report measures but also objective sleep measures (e.g., actigraphy, polysomnography), clinician-rated depression scales, and longer follow-up periods (≥ 6–12 months) to better assess durability of effects and real-world applicability, especially in complex, comorbid inpatient populations. Furthermore, moderator and mediator analysis investigating treatment responses based on various parameters, such as baseline symptom severity or distorted cognitions could provide further insight into which patients are most likely to benefit from ACT treatment. Second, the presented treatment program has demonstrated its feasibility and acceptability within the assessed psychiatric inpatient setting. Patients reported high satisfaction rates and positive feedback regarding the therapeutic alliance, and the benefits, and insights gained from the treatment. Positive experiences with the received treatment are associated with reduced dropout rates, increased adherence, compliance, and persistence, hence contributing to more sustained long-term outcomes (70, 71).

## Limitations

Despite these promising preliminary findings, several limitations need to be considered. First, the presented study comprises of a small sample size (*N* = 8) limiting its generalizability. Although a single-case multiple-baseline design was implemented to assess individual changes over time, large scale RCTs are warranted to increase statistical power, control for possible confounding variables (i.e., improvements could be due to TAU rather than ACT), and assess treatment effectiveness. Specifically, the small sample size increases the influence of individual characteristics on treatment outcomes. Two patients presented with additional psychiatric comorbidities—one with Attention-Deficit/Hyperactivity Disorder and one with Borderline Personality Disorder—which may have shaped symptom trajectories, given that both clinical conditions are associated with greater emotional instability and variable treatment response (72, 73). Moreover, all patients received psychopharmacological treatment during the study, which complicates attribution of improvements solely to ACT. Although medication intake was kept stable for a prolonged period prior to study participation and no medication changes occurred during the intervention phase, pharmacotherapy can independently improve depressive symptoms, while effects on sleep could be heterogeneous (74–76). Thus, while medication stability reduces the likelihood that pharmacotherapy alone accounts for the observed effects, its contribution cannot be ruled out. These sample-related factors further limit generalizability and underscore the need for larger controlled trials allowing stratification by comorbidity and medication status. Second, the study was entirely based on self-report measurements. Incorporating objective measures of sleep quality, as well as clinician-rated scales, such as the Hamilton Depression Rating Scale (77), could provide a more comprehensive assessment of treatment effects, while also mitigating potential biases inherent within self-report measurements. Third, while the assessment time-points chosen provide useful insights into symptomatic change over time before and after receiving ACT, long-term benefits, as well as possible benefits of an add-on booster session post-inpatient treatment to maintain symptom improvement were not evaluated. Third, the ACT intervention was implemented as an add-on to TAU, which itself comprises a multimodal approach including pharmacotherapy, standard CBT, and other therapeutic interventions. This design, while ecologically valid, complicates the interpretation of results. It remains unclear to what extent the observed improvements are attributable solely to ACT-specific processes versus nonspecific therapeutic effects or synergies between ACT and TAU. Although the study attempted to control for these variables by using a multiple baseline design, the absence of an active control condition limits causal inferences. Because ACT was delivered as an add-on to an already multimodal TAU program, the design does not allow clear attribution of improvements to ACT-specific processes. It is possible that TAU alone—or synergies between TAU and ACT—drove a substantial portion of the observed change. Without a TAU-only control condition, causal inferences about ACT’s unique efficacy are not possible. Future studies might therefore consider including an active control group that receives TAU only to better evaluate the unique contributions of ACT. Fourth, another limitation concerns the nature of TAU administered within the inpatient facility. As described, TAU consisted of a comprehensive multimodal program including psychopharmacological treatment, individual CBT-oriented psychotherapy, various behavioral therapy groups, physical activity interventions, and creative therapies. Several of these components overlap conceptually or procedurally with ACT, such as behavioral activation, cognitive distancing techniques, mindfulness and experiential exercises, and engagement in valued or meaningful activities. Because these elements target processes similar to those addressed in ACT—particularly present-moment attention, behavioral engagement, and psychological flexibility—it is not possible to determine the extent to which the observed improvements can be attributed to ACT specifically rather than to TAU, or to possible synergistic effects between the two. This inherent overlap limits interpretability and underscores the need for controlled study designs that isolate ACT mechanisms more clearly. Fifth, another limitation concerns the treatment dose. ACT was delivered in only eight sessions, which may be insufficient for patients with severe depression, particularly given the experiential nature of ACT. More intensive or prolonged intervention formats may produce stronger or more durable effects. Lastly, although all patients fulfilled diagnostic criteria for insomnia disorder, the measurement of insomnia-related outcomes relied exclusively on the Regensburg Insomnia Scale (RIS). While the RIS captures several core symptom dimensions of insomnia—such as prolonged sleep onset latency, reduced sleep duration, difficulties maintaining sleep, or early morning awakening, it does not assess distress due to the sleep disturbance or substantial impairment in daily functioning, which are core symptoms of insomnia disorder according to ICD-10. This discrepancy represents a methodological limitation, as sleep related distress or impairment in daily functioning are central to distinguishing clinical insomnia from nonspecific sleep disturbance. The use of the RIS was based on its strong psychometric properties and its particular sensitivity to cognitive–emotional features of insomnia, which are closely aligned with ACT’s theoretical mechanisms of change. Nevertheless, the absence of a direct assessment of sleep related distress and impairment in functioning means that the present findings primarily reflect changes in insomnia-related symptoms and maladaptive sleep cognitions rather than comprehensive insomnia disorder severity. Future studies should therefore integrate validated measures that explicitly assess sleep dissatisfaction to more fully capture diagnostic criteria and to enhance interpretability in accordance with contemporary insomnia research.

## Conclusion

In sum, the present study provides preliminary evidence for the efficacy of ACT as an adjunct treatment for inpatients diagnosed with severe depressive disorder and comorbid insomnia. Significant improvements in depressiveness, as well as sleep-related difficulties, along with improved acceptance of sleep-related problems, psychological flexibility, and overall physical and psychological well-being over time provide support for the benefits of ACT as a transdiagnostic tool addressing complex and multimorbid psychiatric conditions. However, due to several limitations—i.e., small sample size, self-report measures only, confounding factors due to simultaneous TAU—results need to be interpreted with caution. Therefore, future research should address these drawbacks by incorporating large-scale RCTs, including objective and clinician-rated scales, in order to validate the presented preliminary findings, increase reliability, and assess treatment effectiveness. Nevertheless, the presented findings can inform clinical research and practice, and provide initial insights into the development of more comprehensive and sustainable transdiagnostic treatment programs, which could ultimately contribute to long-term symptomatic improvement for patients suffering from depression and comorbid sleep-related difficulties.

## Data Availability

The raw data supporting the conclusions of this article will be made available by the authors, without undue reservation.
